# Effect of Integrated Extraction Techniques on the Technofunctional and Bioactive Properties of *Brosimum alicastrum* Swartz Proteins

**DOI:** 10.3390/foods13182875

**Published:** 2024-09-11

**Authors:** María Fernanda Suárez-Hernández, Sara Gabriela Posada Ramirez, Darling del Carmen Castillo Cruz, Inocencio Higuera Ciapara, Neith Aracely Pacheco López, Iván Emanuel Herrera Pool, Jorge Carlos Ruiz-Ruiz

**Affiliations:** 1 School of Nutrition (Mérida), Universidad Anáhuac, Avenida Universidad Anáhuac No. 46, Col. Lomas Anáhuac, Huixquilucan C.P. 52786, Estado de México, Mexicoinocencio.higuera@anahuac.mx (I.H.C.); 2 Universidad Politécnica de Yucatán, Tablaje Catastral 7193, Carretera Mérida-Tetiz Km 4.5, Ucú C.P. 97357, Yucatán, Mexico; 3 Centro de Investigación y Asistencia en Tecnología y Diseño del Estado de Jalisco, A.C., Subsede Sureste, Parque Científico Tecnológico de Yucatán, Km 5.5 Sierra Papacal-Chuburná Puerto, Mérida C.P. 97302, Yucatán, Mexico

**Keywords:** plant-based proteins, extraction, synergism, functional properties, biological activities

## Abstract

This study addresses the need for effective protein extraction and characterization to unlock the potential of underutilized plant resources like *Brosimum alicastrum* Swartz nuts, aiming to enhance their value as functional ingredients in food applications. Extraction methods, including pH modulation, ultrasound-assisted extraction, and enzymatic hydrolysis, are employed to enhance technofunctional and bioactive properties. The protein extracts are evaluated for solubility, emulsifying capacity, foaming properties, and water/oil-holding capacities to assess their technofunctional potential. Additionally, the bioactive properties, such as antioxidant and anti-inflammatory activities, are analyzed to explore potential health benefits. The results demonstrate that integrated extraction techniques significantly improve the yield and quality of *Brosimum alicastrum* Swartz nut proteins. Enzymatic hydrolysis, in particular, produces hydrolysates with superior bioactive properties. These findings highlight the potential of *Brosimum alicastrum* Swartz proteins as valuable ingredients for the food and pharmaceutical industries, promoting the utilization of underexploited plant resources for sustainable and health-promoting applications.

## 1. Introduction

In the quest for sustainable and nutritious food sources, *Brosimum alicastrum* Swartz, commonly known as the Maya nut or breadnut, has emerged as a promising candidate. Native to the tropical forests of Central and South America, *Brosimum alicastrum* seeds are rich in proteins (12–16%), essential amino acids (lysine: ~6–7 g/100 g of protein, methionine: ~1–2 g/100 g of protein, tryptophan: ~0.5–1 g/100 g of protein), and a variety of vitamins (vitamin A ~50 µg/100 g and vitamin C ~20 mg/100 g) and minerals (calcium ~100 mg/100 g, potassium ~700 mg/100 g, and iron ~5 mg/100 g), so they could be used as an alternative for those seeking plant-based protein sources [1]. The cultivation of *B. alicastrum* Swartz aligns with sustainable agricultural practices, requiring minimal inputs and exhibiting resilience to pests and diseases. The commercialization of *B. alicastrum* Swartz offers economic opportunities by the development of value-added products, such as protein-rich flours, protein isolates and concentrates, and hydrolysates [2]. The development of innovative food technologies can expand the use of *B. alicastrum* nut proteins, catering to the growing demand for plant-based alternatives.

Plant-based proteins are increasingly recognized for their health benefits, environmental sustainability, and potential to meet the dietary needs of a growing population. Traditional methods of extracting plant-based proteins have been used for decades. These techniques typically involve grinding plant materials, followed by the use of solvents (alkaline or acidic aqueous solutions or water–alcohol mixtures) or physical force to separate the proteins from other components [3]. While effective, these methods often result in lower yields, potential loss of nutritional value (denaturation, loss of biological activity, loss of amino acids), and environmental concerns. Integrated extraction techniques like enzymatic hydrolysis, ultrafiltration, supercritical fluids, and others aim to overcome the limitations of traditional methods by combining various technologies to maximize yield, enhance quality, and reduce environmental impact [4]. The proteins extracted using integrated techniques have diverse applications in the food industry and can be used in the formulation of meat substitutes, dairy alternatives, protein supplements, and functional foods. Additionally, these high-quality plant proteins can cater to the growing market demand for clean-label, non-GMO, and organic products [5].

By synergistically integrating protein extraction techniques such as pH modulation, ultrasound-assisted extraction, and enzymatic hydrolysis, it would be feasible to obtain higher protein yields, improved protein quality, an eco-friendly process, and lower overall production costs [6]. Additionally, the technofunctional and bioactive properties of the extracted proteins could be improved [7]. Considering the above arguments, the aim of this study is to determine the synergistic effect of pH modulation, ultrasound-assisted extraction, and enzyme-assisted aqueous extraction on the technofunctional and bioactive properties of *Brosimum alicastrum* Swartz nut proteins.

## 2. Material and Methods

### 2.1. Materials

Raw flour of *Brosimum alicastrum* seeds was provided by the Natural Resources Unit of the Yucatan Scientific Research Centre. All reagents used were analytical grade and purchased from J.T. Baker (Phillipsburg, NJ, USA), Sigma (Sigma Chemical Co., St. Louis, MO, USA), Merck (Darmstadt, Germany), and Bio-Rad (Bio-Rad Laboratories, Inc. Hercules, CA, USA).

### 2.2. Protein Extraction

pH modulation. Initially, 50 g of flour was dispersed in 500 mL of distilled water (1:10, *w*/*v*) and stirred for 30 min at ambient temperature. The pH of the flour suspension was adjusted to the required pH 12 using 1.0 M NaOH and stirred for 1 h.

Ultrasound-assisted extraction. Flour suspension at pH 10 was placed in a vessel suitable for ultrasonic processing and extracted at a frequency of 25 kHz and a power 400 W for 30 min and 50 °C to prevent protein denaturation.

Enzyme-assisted aqueous extraction. For enzyme-assisted aqueous extraction the following proteases were used: *Bacillus subtilis* protease, *Bacillus amyloliquefaciens* protease, and *Bacillus licheniformis* protease. The extractions were carried out as follows: flour suspension was adjusted to pH 7, temperature was at 50 °C, enzyme–substrate ratio was 1.5% *w*/*v*, with constant stirring (150 rpm), for 10, 20, and 30 min. Then extracts were adjusted to pH 4.3 to precipitate proteins and centrifuged at 3000× *g* for 15 min at room temperature to remove the liquid fraction. Finally, precipitates were lyophilized and preserved at −18 °C until analysis. Thus, extraction treatment 1 (ET1) = pH 10, ultrasound-assisted extraction (25 kHz, 400 W, 30 min, 50 °C) and enzyme-assisted aqueous extraction with *Bacillus subtilis* protease. Extraction treatment 2 (ET2) = pH 10, ultrasound-assisted extraction (25 kHz, 400 W, 30 min, 50 °C) and enzyme-assisted aqueous extraction with *Bacillus amyloliquefaciens* protease. Extraction treatment 3 (ET3) = pH 10, ultrasound-assisted extraction (25 kHz, 400 W, 30 min, 50 °C) and enzyme-assisted aqueous extraction with *Bacillus licheniformis* protease.

### 2.3. Characterization of Technofunctional Properties

#### Water Protein Solubility (WPS)

This was determined according to the method reported by Rodriguez-Ambriz [8] with some modifications. First, 50.0 mg of protein extracted at different pH values was dispersed in 5.0 mL of distilled water. The mixture was stirred at room temperature for 10 min and centrifuged at 3000× *g* rpm for 20 min. Protein contents in the supernatant were determined using the Bradford method [9]. All analyses were performed in triplicate. Protein solubility was then calculated using the following formula:

Solubility (%) = (Protein content in supernatant/Total protein content in sample) × 100

### 2.4. Emulsifying Properties

Protein extracted at different pH values (50 mg) was homogenized at 7000 rpm for 1 min in 25 mL distilled water. The protein solution was mixed with 25 mL of soybean oil followed by homogenization at 7000 rpm for 1 min. The emulsion was centrifuged at 3000× *g* rpm for 5 min. The EC was calculated using the following formula [10]:

EC (%) = (Volume of emulsified layer/Total volume) × 100

Emulsion stability (ES) was determined by centrifugation at 3000× *g* rpm for 5 min followed by heating at 80 °C for 30 min and then calculated as follows:

ES (%) = (Volume of remaining layer/Total volume) × 100

### 2.5. Foaming Properties

Foaming capacity (FC) and stability (FS) were determined according to the method described by Sze-Tao and Sathe [11] with some modifications. First, 50 mg of protein extracted at different pH values was dispersed in 50 mL of distilled water. Solutions were stirred at 7000 rpm for 2 min. The blend was transferred into a 100 mL graduated cylinder. The volume was recorded before and after stirring. For the determination of FS, foam volume changes in the graduated cylinder were recorded at 30 min of storage. All analysis was performed in triplicate. The FC and FS were then calculated according to the following formula:

FC (%) = [(volume after wiping − volume before whipping)/volume before whipping] × 100

FS (%) = [(volume after standing − volume before whipping)/volume before whipping] × 100

### 2.6. Fat Absorption Capacity (FAC)

The FAC was determined using the method described by Kaur and Singh [12] with some modifications. First, 1.0 g of protein extracted at different pH values was weighed into a 15 mL pre-weighed centrifuge tube and mixed with 5.0 mL soybean oil. After being held for 30 min the emulsion was centrifuged at 3000× *g* rpm for 20 min. Then the supernatant was removed, and the tube was weighed. All analyses were performed in triplicate. FAC was calculated using the following formula:

FAC (g of oil/g of sample) = (F_2_ − F_1_)/F_0_

where F_0_ is the weight of the dry sample (g), F_1_ is the weight of the tube plus the dry sample (g), and F_2_ is the weight of the tube plus the sediment (g).

### 2.7. Water Absorption Capacity (WAC)

The WAC was determined using the method described by Rodriguez-Ambriz [8] with some modifications. First, 1.0 g of protein extracted at different pH values was weighed into a 15 mL pre-weighed centrifuge tube. Then 10 mL of distilled water was added. After being held for 30 min, the tube was centrifuged at 2000× *g* rpm for 20 min. All analyses were performed in triplicate. The WAC was calculated using the following formula:

WAC (g of water/g of sample) = (W_2_ – W_1_)/W_0_

where W_0_ is the weight of the dry sample (g), W_1_ is the weight of the tube plus the dry sample (g), and W_2_ is the weight of the tube plus the sediment (g).

### 2.8. Amino Acid Content and Distribution

Hydrolyzed amino acids: For the extraction of amino acids from the protein fraction of samples, 50.0 mg was added to 10 mL amber containers. Then, 3.0 mL of 6.0 M HCl was added to the samples and the containers were hermetically sealed with a press. The extraction was carried out at 110 °C for a period of 24 h. After completing the hydrolysis, the samples were partially neutralized with 2.5 mL of 4.0 M NaOH. Subsequently, the samples were filtered with 0.45 µm membrane filters (PTFE). The recovered material was frozen at −40 °C until analysis.

a. Extraction of free amino acids. For the extraction of amino acids, approximately 50.0 mg of material was weighed into 15.0 mL conical tubes. Then, 10.0 mL of type II water was added. The extraction was carried out in a sonicator bath (Bransonic Ultrasonic Cleaner; Model: 3510R-MT; Danbury, CT, USA) for a period of 60 min. After finishing, the samples were centrifuged in a centrifuge (refrigerated centrifuge; Thermoscientific; model: SORVALL ST 8R; Waltham, MA, USA) at 4500× *g* rpm for 15 min at 4 °C and the supernatant was filtered with 0.45 µm membrane filters (PTFE). The recovered material was frozen at −40 °C until analysis.

b. Derivatization. For derivatization, 250 µL of liquid sample was placed in a 15 mL conical tube. Then, 15 µL of HCl (37% *v*/*v*) was added, 500 µL of acetonitrile, and 1.5 mL of 0.05 M borate buffer. Subsequently, 30 µL of 10 M NaOH and 1.25 mL of derivatization agent were added; then FMOC-Cl at a concentration of 1.5 mg/mL. Derivatization was performed at 60 °C for 30 min. At the end, 150 µL of formic acid (88% purity) was added. Samples were filtered with 0.22 µm membrane filters (PTFE) and placed in chromatographic vials for analysis by UPLC-ESI-MS/MS. The samples and calibration curve points were derivatized with the same procedure.

c. Analysis using UPLC-ESI-MS/MS. The determination of amino acids was performed using UPLC-ESI-MS/MS.

d. Chromatographic parameters. Chromatographic profiles were obtained using a Waters Acquity H Class UPLC (Milford, MA, USA) with a quaternary pump (UPQSM) and an autosampler (UPPDALTC) coupled to a Waters Xevo-TQ micro mass spectrometer. Column: chromatographic separation was performed with a Waters Acquity UPLC AccQ-TagT Ultra 1.7 µm column, 100 × 2.1 mm ID (Milford, MA, USA). Column temperature: 60 °C. Mobile phase: 10% acetonitrile with 0.1% *v*/*v* formic acid and 0.05 mM ammonium acetate was used as mobile phase A; phase B was treated with acetonitrile (0.1% *v*/*v* formic acid).

### 2.9. In Vitro Antioxidant Properties

#### Free Radical Scavenging

The radical-scavenging capacity on 1,1-diphenyl-2-picrylhydrazyl (DPPH) free radicals was measured as described by Diniyah [13]. A volume of 500 μL (100 mg/mL) of protein extracted at different pH values was mixed with 500 μL of 0.1 mM DPPH (95% ethanol). The mixture was homogenized and left to react for 30 min at room temperature. Absorbance was determined at 517 nm. The percentage of the DPPH free radical scavenging was calculated using the following equation:

Radical-scavenging capacity (%) = ((A_0_ − A_1_)/A_0_) × 100

where A_0_ was the absorbance of the blank and A_1_ was the absorbance in the presence of protein extracts. The IC50 (concentration providing 50% radical scavenging) values were calculated using the dose inhibition curve in the linear range by plotting the sample concentration versus the corresponding scavenging effect. Ascorbic acid was used as a control.

### 2.10. Chelating Capacity

Chelating capacity was determined using the pyrocatechol violet reagent according to Niveditha and Sridhar [14]. Briefly, 1.0 mL of sodium acetate buffer (100 mM, pH 4.9), 100 μL of Cu (II) standard solution (1.0 mg/mL), and 100 μL (100 mg/mL) of protein extracted at different pH values were homogenized in a test tube. The mixture reacted for 5 min at room temperature and 25 μL of a pyrocatechol violet solution (4.0 mmol/L) was then added. Absorbance was determined at 632 nm. The chelating activity was calculated as follows:

Chelating capacity (%) = (1 − sample absorbance/blank absorbance) × 100.

The IC50 (concentration providing 50% chelating effect) values were calculated using the dose inhibition curve in a linear range by plotting the protein extract concentration versus the corresponding scavenging effect. Ascorbic acid was used as a control.

### 2.11. Reducing Power

This method is based on the reduction of potassium ferricyanide (Fe^3+^) in the presence of an antioxidant to (Fe^2+^) forming the blue complex K[Fe^II^(CN_6_)], which absorbs at 700 nm [13]. First, 200 μL (100 mg/mL) of protein extracted at different pH values, 500 μL of phosphate buffer (0.2 M, pH 6.6), and 500 μL of potassium ferricyanide (1%) were homogenized in a test tube. The test tube was then incubated at 50 °C for 20 min. Subsequently, 500 μL of trichloroacetic acid 10% (*w*/*v*) was added, and the tube was centrifuged at 3000× *g* for 10 min. An aliquot of 500 μL of the supernatant was dissolved in an equal amount of distilled water and, immediately, 500 μL of ferric chloride (0.1%) was added. Absorbance was determined at 700 nm. The percentage inhibition of the K[Fe^II^(CN_6_)] complex formation was calculated as:

Reducing power = [(A_0_ − A_1_)/A_0_] × 100

where A_0_ was the absorbance of the control and A_1_ of the mixture containing the extract or the absorbance of a protein fraction. The IC50 (concentration providing 50% reducing power) values were calculated using the dose inhibition curve in a linear range by plotting the protein extract concentration versus the corresponding scavenging effect. Ascorbic acid was used as a control.

### 2.12. In Vitro Anti-Inflammatory Properties

#### Inhibition of Protein Thermal Denaturation

The method of Khan [15] was followed with minor modifications. The reaction mixture consisted of protein (100 mg/mL) extracted at different pH values and a 1% aqueous solution of ovalbumin. The pH of the reaction was adjusted to 7.0 using a small amount of 1.0 mol/L HCl. A volume of 100 μL (100 mg/mL) of protein extracted at different pH values and a volume of 100 μL of 1% aqueous solution of ovalbumin were incubated at 37 °C for 20 min and then heated at 57 °C for 20 min. After cooling the samples, the turbidity was measured spectrophotometrically at 660 nm. The experiment was performed in triplicate. Percent inhibition of protein denaturation was calculated as follows:

Inhibition of albumin denaturation (%) = (Abs blank − Abs sample) × 100/Abs blank.

### 2.13. Cell Membrane Stabilization

Fresh whole human blood (10 mL) was collected and transferred to heparinized centrifuged tubes. The tubes were centrifuged at 3000× *g* rpm for 10 min and were washed three times with an equal volume of isosaline. The volume of the blood was measured and reconstituted as a 10% *v*/*v* suspension with isosaline [16]. The reaction mixture (700 μL) consisted of 350 μL (100 mg/mL) of proteins extracted at different pH values and 350 μL of 10% of red blood cell suspension and, instead of a sample, only isosaline solution was added to the blank test tube. All the centrifuge tubes containing the reaction mixture were incubated in a water bath at 56 °C for 30 min. At the end of the incubation, the tubes were cooled under running tap water. The reaction mixture was centrifuged at 2500× *g* rpm for 5 min and the absorbance of the supernatant was taken at 560 nm. The experiment was performed in triplicates. Percent membrane stabilization activity was calculated by the following formula:

Membrane stability = 100 − [100 × (Protein extract Abs − Control Abs)/Control Abs]

### 2.14. Statistical Analysis

Results are reported as mean ± standard errors of the mean. An independent samples *t*-test was used for comparison between groups. Differences were considered significant at *p* < 0.05. XLSTAT software was used.

## 3. Results and Discussion

### 3.1. Characterization of Technofunctional Properties

**Figure 1:**
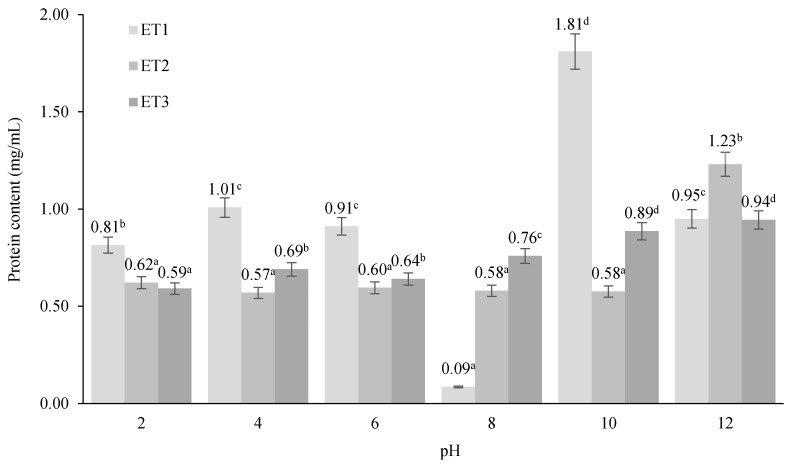
Effect of integrated extraction techniques on protein solubility (mg/mL) of *Brosimum alicastrum* Swartz nut proteins at different pH values. ET1 (pH 10, 25 kHz, 400 W, 30 min, 50 °C, and *B. subtilis* proteases); ET2 (pH 10, 25 kHz, 400 W, 30 min, 50 °C, and *B. amyloliquefaciens* proteases); and ET3 (pH 10, 25 kHz, 400 W, 30 min, 50 °C, and *B. licheniformis* proteases). Different letters indicate significant differences (*p* < 0.05).

Results of protein solubility determination in water are presented in Figure 1. Protein content varies significantly across different pH levels for all three treatments. The highest protein content is observed in ET1 at pH 10 (1.81 mg/mL), which indicates that this pH is optimal for protein extraction or solubility in ET1. Conversely, the lowest protein content is observed in ET1 at pH 8 (0.09 mg/mL), suggesting that this pH is unfavorable for protein extraction in ET1. The significant spike in protein content for ET1 at pH 10 suggests a unique property or component in ET1 that enhances protein solubility or extraction at this pH. At pH 10 and 12, ET1 continues to show high protein content compared to ET2 and ET3, but the differences are less pronounced compared to pH 10.

Results indicate that the pH of a technofunctional assay has a significant effect on the soluble protein content for the different extraction treatments. In the case of ET1, the protein content increases and remains stable when passing from pH 2 to pH 4 and pH 6, showing a significant decrease at pH 8 to increase to a maximum at pH 10, and then decreasing again to a content similar to that reached at pH values of 4 and 6. Comparatively, ET2 and ET3 maintain stable protein concentrations throughout the pH range evaluated (2–10), with both treatments showing their maximum concentrations at pH 12. The increase in protein content at higher pH values (10 and 12) for all treatments suggests that alkaline conditions are favorable for protein solubility or extraction.

Protein solubility is a critical technofunctional property that influences the performance of proteins in various food applications [17]. Results suggest that ET1, with its higher solubility at pH 10, may offer superior functionality in emulsification, foaming, gelation, and water-binding applications. Conversely, ET2 and ET3 might be more suitable for applications where lower solubility is beneficial. Adjusting the pH can be a critical tool in maximizing the desired functional properties of proteins in various food applications.

Table 1 shows the effect of integrated extraction techniques on emulsifying capacity (EC), emulsion stability (ES), foam capacity (FC), and foam stability (FS) of *Brosimum alicastrum* Swartz nut proteins at different pH values. For emulsifying capacity (EC) and emulsion stability (ES), ET1 shows high emulsifying capacity across all pH levels, with maximum activity at pH 4 to 12. However, emulsion stability is only detected at pH 4 to 10. ET2 exhibits similar high emulsifying activity but with slightly higher emulsion stability at pH 12. ET3 has the lowest emulsifying activity and stability at lower pH levels but matches the other treatments at higher pH. Results suggest that ET1 and ET2 proteins are excellent emulsifiers across a broad pH range, while ET3 is less effective, particularly at lower pH values.

For foam capacity (FC) and foam stability (FS), ET1 shows consistent foam capacity and stability at pH 2, 4, and 6, but no detection at pH 8 and 10, with minimal activity at pH 12. ET2 and ET3 have no foam capacity or stability at pH 2 and 4. They exhibit similar behavior at pH 6 and 8 but have higher values at pH 10 and 12, with ET2 showing higher foam capacity than ET3 at pH 12. Results indicate that ET1 proteins maintain their foaming properties better at lower pH, while ET2 and ET3 perform better at higher pH. The emulsifying and foaming capacities of proteins are critical technofunctional properties that significantly influence the quality and stability of various food products [18]. The data suggest that ET1 proteins are particularly effective in lower-pH environments for foaming applications and across a wide pH range for emulsifying applications. ET2 and ET3 are more effective at higher pH levels, making them suitable for specific applications where such conditions are prevalent.

Table 2 presents the effect of integrated extraction techniques on fat and water absorption capacities of *Brosimum alicastrum* Swartz nut proteins for three different treatments. For fat absorption capacity, ET1 increases from pH 2 to 6, peaks at pH 6 (4.8%), and then decreases. ET2 follows a similar pattern with a peak at pH 6 (4.8%) and slightly lower values than ET1. ET3 shows the lowest fat absorption capacity values, peaking at pH 6 (4.2%). Higher fat absorption capacity is beneficial for products like sausages, meat patties, and baked goods where fat retention enhances flavor and texture [18]. ET1 and ET2 at pH 6 are most advantageous for these uses.

For water absorption capacity (WAC), ET1 shows increasing WAC from pH 2 to 6, peaking at pH 6 (4.5%) and then gradually decreasing towards pH 12. ET2 exhibits similar trends with a peak at pH 6 (4.0%), but generally higher values than ET1 and ET3 at lower pH levels. ET3 also increases to a peak at pH 6 (3.5%) but has the lowest WAC values among the three treatments across all pH levels. Higher WAC is desirable in products like meat substitutes, baked goods, and sauces, where retaining moisture is critical for texture and mouthfeel [19]. ET1 and ET2 at pH 6 would be most suitable for such applications.

Although alkaline treatment is time- and energy-consuming, and its alkaline–acid solvents may reduce protein quality, this treatment has the advantage that it can be adapted to any source of vegetable protein, obtaining good recovery yields without affecting the technofunctional properties [20]. On the other hand, ultrasound-assisted extraction when combined with conventional techniques significantly improves recovery yields and by properly selecting the conditions of frequency, intensity, time, and temperature, the quality of the proteins is not affected [21]. Finally, during enzyme-assisted extraction, proteases fractionate high-molecular-weight proteins into smaller and more soluble portions, favoring the extraction conditions. Additionally, they work under optimal pH conditions, avoiding protein denaturation [22]. In general, the combined treatment of a conventional method of alkaline extraction with ultrasound-assisted extraction and enzyme-assisted extraction exhibited a significant effect on the technofunctional properties of *B. alicastrum* nut proteins.

Results indicate that the highest concentrations of soluble protein were obtained with ET1 extraction treatment, followed by ET3 and ET2. For the capacity and emulsifying stability, ET1 presented the highest level of processes, followed by ET2 and ET3. The foaming capacity and stability did not present consistent data and in some cases this property was not detected. For water absorption capacity, ET1 and ET2 presented the highest values and for oil absorption the highest value was for the ET3 extraction treatment. From the extraction methods described above, the method assisted by enzymes stands out because it can generate fragments with specific sequences that could most significantly influence the technofunctional properties. The above is corroborated with the results obtained, highlighting the treatment ET1 that uses proteases of *B. subtilis*, which could have generated peptides with hydrophilic characteristics that influence the technofunctional properties [23].

### 3.2. Amino Acid Content and Distribution

The amino acid content and distribution in proteins extracted from *Brosimum alicastrum* Swartz nuts using different integrated extraction techniques (ET1, ET2, ET3) provide insights into their potential antioxidant and anti-inflammatory properties (Table 3). The distribution of amino acids affects the overall functionality of the protein extracts, which can influence their antioxidant and anti-inflammatory properties. Nonpolar or hydrophobic amino acids are crucial for protein folding and stability. ET2 has the highest percentage (15.01%), followed by ET1 (14.50%) and ET3 (14.08%). This suggests that ET2 might have better structural integrity and stability, potentially enhancing its functional benefits. In the case of uncharged polar and negatively charged amino acids, these amino acids are involved in catalytic activity and interactions with other molecules, influencing both antioxidant and anti-inflammatory activities. The distribution percentages are relatively similar among the three extraction methods, with ET3 showing a slightly higher percentage of negatively charged amino acids (34.51%).

ET1 and ET2 demonstrate higher contents of key amino acids associated with antioxidant activity, such as glutamic acid and glycine. This suggests that proteins extracted using *B. subtilis* and *B. amyloliquefaciens* proteases may offer better protection against oxidative stress. ET1 and ET2 also show higher levels of amino acids known for their anti-inflammatory effects, such as arginine and branched-chain amino acids (leucine, isoleucine, and valine). This indicates that these extraction techniques might be more effective in mitigating inflammation. The consistently lower amino acid content in ET3 suggests that *B. licheniformis* proteases are less effective in extracting proteins with high antioxidant and anti-inflammatory potential. This could be due to differences in protease activity or protein degradation during extraction.

### 3.3. In Vitro Antioxidant Properties

Table 4 presents the effect of integrated extraction techniques on in vitro antioxidant properties of *Brosimum alicastrum* Swartz nut proteins for three different treatments. Free-radical-scavenging activity of *Brosimum alicastrum* Swartz nut proteins varied significantly across the three extraction techniques. ET2 exhibited the highest radical-scavenging activity with 42.56%, followed by ET1 and ET3, with values of 33.19% and 32.16%, respectively. The significant difference in radical-scavenging activity among the ETs suggests that the type of protease used in the extraction process influences the antioxidant properties of the proteins. ET2, using *B. amyloliquefaciens* proteases, enhanced the radical-scavenging activity more effectively than the other proteases. Free-radical-scavenging ability of proteins is a vital property that significantly influences the quality, safety, and shelf life of protein-based products and foods. Incorporating antioxidants in these products can lead to better preservation of sensory attributes, nutritional value, and overall product stability [24].

Chelating capacity, which indicates the ability to bind metal ions, also showed significant differences among the three extraction techniques. ET3 demonstrated the highest chelating capacity with 52.94%, followed by ET1 (25.78%) and ET2 (18.73%). This suggests that the *B. licheniformis* proteases used in ET3 were more effective in enhancing the chelating capacity of the *B. alicastrum* nut proteins. The increased chelating capacity of ET3 could be beneficial in applications where metal ion binding is crucial for antioxidant activity. By binding metal ions, chelating agents prevent metal-catalyzed oxidative damage, enhance the nutritional quality by improving mineral bioavailability, and ensure food safety by reducing the risk of toxic metal contamination and inhibiting microbial growth. The use of chelating agents in the food industry is essential for developing high-quality, stable, and nutritious protein-based products [25].

The reducing power of the nut proteins, which measures their electron-donating ability, varied significantly with the extraction method. ET1 had the highest reducing power with 85.31%, followed by ET2 with 73.08% and ET3 with 53.43%. This indicates that the proteases from *B. subtilis* used in ET1 were most effective in enhancing the reducing power of the nut proteins. The high reducing power of ET1 suggests its potential application in food systems where reducing agents are required to prevent oxidation. By donating electrons to neutralize free radicals and prevent oxidative damage, reducing agents help preserve the sensory attributes, nutritional value, and overall stability of these products. Their application in the food industry is essential for developing high-quality, long-lasting protein-based foods [26].

Results demonstrated that different extraction techniques significantly influenced the antioxidant properties of *Brosimum alicastrum* Swartz nut proteins. The type of protease used in the extraction process significantly affects the antioxidant properties of the extracted proteins. This highlights the importance of selecting appropriate proteases based on the desired antioxidant activity in food applications. Findings suggest that *Brosimum alicastrum* Swartz nut proteins, with optimized antioxidant properties through specific extraction techniques, can be effectively used in various food and nutraceutical products to enhance their shelf life, nutritional value, and functional properties.

### 3.4. In Vitro Anti-Inflammatory Properties

Table 5 shows the effect of integrated extraction techniques on inhibition of protein thermal denaturation and cell membrane stabilization of *Brosimum alicastrum* Swartz nut proteins. Protein thermal denaturation is a process where proteins lose their tertiary structure due to the application of heat, leading to loss of function. The ability to inhibit this process is indicative of the anti-inflammatory potential of the proteins [27]. ET1 showed an inhibition of 40.44%. This is the lowest inhibition among the three techniques, suggesting that the proteins extracted using *B. subtilis* proteases are less effective in preventing protein denaturation under thermal stress. ET2 demonstrated the highest inhibition at 64.11%. This significant increase indicates that *B. amyloliquefaciens* proteases are more effective in extracting proteins that can prevent thermal denaturation. Finally, ET3 had an inhibition value of 57.35%, which is significantly higher than ET1 but lower than ET2. This suggests that *B. licheniformis* proteases are more effective than *B. subtilis* proteases but less effective than *B. amyloliquefaciens* proteases. The significant differences between the extraction techniques (*p* < 0.05) highlight the impact of the type of protease used on the anti-inflammatory properties of the extracted proteins. ET2, using *B. amyloliquefaciens* proteases, appears to be the most effective in enhancing protein stability against thermal denaturation.

Cell membrane stabilization is another measure of anti-inflammatory activity, reflecting the ability to protect cells from lysing under stress [16]. ET1 exhibited cell membrane stabilization at 4.78%. This lower value indicates a relatively weaker ability to stabilize cell membranes compared to the other methods. ET2 showed an improved stabilization effect with a value of 7.73%. This significant enhancement suggests that the proteins extracted with *B. amyloliquefaciens* proteases can better protect cell membranes. Finally, ET3 demonstrated a stabilization value of 5.94%, which is significantly better than ET1 but lower than ET2. This indicates that the proteins extracted using *B. licheniformis* proteases offer moderate protection to cell membranes. Results again show significant differences between the extraction techniques, with ET2 providing the highest cell membrane stabilization, followed by ET3 and ET1. This trend mirrors the inhibition of protein thermal denaturation, indicating that the protease type used in extraction significantly influences both anti-inflammatory properties.

The incorporation of proteins with antioxidant and anti-inflammatory properties into foods and products offers substantial health benefits, from reducing the risk of chronic diseases to enhancing the functional and sensory qualities of food products. As consumer interest in health and wellness continues to grow, leveraging these properties can drive innovation and provide competitive advantages in the food industry. Future studies could explore the underlying mechanisms by which different proteases affect protein functionality and further optimize extraction conditions to maximize antioxidant and anti-inflammatory benefits.

## 4. Conclusions

The present study provides a comprehensive analysis of the technofunctional, antioxidant, and anti-inflammatory properties of *Brosimum alicastrum* Swartz nut proteins extracted using three different integrated extraction techniques (ET1, ET2, ET3). ET1 (*B. subtilis* proteases) and ET2 (*B. amyloliquefaciens* proteases) generally offer superior technofunctional properties compared to ET3 (*B. licheniformis* proteases). In terms of bioactivity, the three extraction treatments generated antioxidant activity through multiple mechanisms. ET1 presented the highest percentage of reducing power, ET2 exhibited the greatest value for free radical scavenging, while ET3 had the highest percentage of the chelating capacity of prooxidant cations. On the other hand, anti-inflammatory activity of ET2 exhibited the greatest bioactivity in the two in vitro trials, inhibition of protein thermal denaturation and cell membrane stabilization, followed by ET3 and ET1. Considering both technofunctional properties and bioactivity, ET1 extraction treatment could be used when solubility, emulsifying properties, water absorption capacity, and a combination of antioxidant and anti-inflammatory capacities are required. Meanwhile, ET2 and ET3 extraction treatments could be applied in food systems that require less solubility, oil absorption capacity, and a combination of antioxidant and anti-inflammatory capacities. The proteins derived from *Brosimum alicastrum* Swartz nuts are highly relevant for both health and functional food applications. Their rich amino acid profile and antioxidant and anti-inflammatory properties, along with excellent technofunctional properties, position them as valuable ingredients in the growing market for plant-based proteins and functional foods. Optimizing extraction techniques to maximize these benefits can drive innovation and offer competitive advantages in the food and nutraceutical industries. Future research and development should focus on further exploring these proteins’ potential and developing products that can deliver their health benefits effectively.

## Figures and Tables

**Table 1:** Effect of integrated extraction techniques on foam capacity (%), foam stability (%), emulsifying activity (%), and emulsion stability (%) of *Brosimum alicastrum* Swartz nut proteins at different pH values.

Extraction Treatment	ET1		ET2		ET3		ET1		ET2		ET3	
pH	FC	FS	FC	FS	FC	FS	EC	ES	EC	ES	EC	ES
2	20.0 ^a^	20.0 ^a^	ND	ND	ND	ND	50.0 ^a^	ND	55.6 ^a^	ND	44.4 ^a^	ND
4	ND	ND	ND	ND	20.0 ^a^	20.0 ^a^	60.0 ^b^	10.0 ^a^	55.6 ^a^	ND	44.4 ^a^	11.1
6	20.0 ^a^	20.0 ^a^	20.0 ^a^	20.0 ^a^	ND	ND	100.0 ^c^	10.0 ^a^	100.0 ^b^	10.0 ^a^	60.0 ^b^	10.0 ^a^
8	ND	ND	ND	ND	ND	ND	100.0 ^c^	20.0 ^b^	100.0 ^b^	10.0 ^a^	100.0 ^c^	20.0 ^b^
10	ND	ND	20.0 ^a^	20.0 ^a^	40.0 ^b^	40.0 ^b^	100.0 ^c^	20.0 ^b^	100.0 ^b^	20.0 ^b^	100.0 ^c^	20.0 ^b^
12	20.0 ^a^	ND	ND	ND	ND	ND	100.0 ^c^	20.0 ^b^	100.0 ^b^	20.0 ^b^	100.0 ^c^	30.0 ^c^

ET1 (pH 10, 25 kHz, 400 W, 30 min, 50 °C, and *B. subtilis* proteases); ET2 (pH 10, 25 kHz, 400 W, 30 min, 50 °C, and *B. amyloliquefaciens* proteases); and ET3 (pH 10, 25 kHz, 400 W, 30 min, 50 °C, and *B. licheniformis* proteases). Different letters in the same column indicate significant differences (*p* < 0.05). ND = Not detected.

**Table 2:** Effect of integrated extraction techniques on water absorption capacity (WAC, 1 g of water/g of protein) and fat absorption capacity (FAC, 1 g of oil/g of protein) of *Brosimum alicastrum* Swartz nut proteins.

Extraction	WAC	FAC
ET1	3.4 ± 0.2 ^b^	2.2 ± 0.1 ^a^
ET2	3.3 ± 0.1 ^b^	2.4 ± 0.2 ^a^
ET3	2.7 ± 0.1 ^a^	2.8 ± 0.1 ^b^

ET1 (pH 10, 25 kHz, 400 W, 30 min, 50 °C, and *B. subtilis* proteases); ET2 (pH 10, 25 kHz, 400 W, 30 min, 50 °C, and *B. amyloliquefaciens* proteases); and ET3 (pH 10, 25 kHz, 400 W, 30 min, 50 °C, and *B. licheniformis* proteases). WAC = Water absorption capacity. FAC = Fat absorption capacity. Different letters in the same column indicate significant differences (*p* < 0.05).

**Table 3:** Effect of integrated extraction techniques on amino acid content (g of amino acid/kg of sample) and distribution (%) by UPLC-ESI-MS/MS of *Brosimum alicastrum* Swartz nut proteins.

Amino Acid	ET1	ET2	ET3
Alanine	2.06	1.85	0.23
Arginine	1.89	1.70	0.20
Aspartic acid	2.16	1.81	0.28
Cysteine	ND	ND	ND
Glutamic acid	3.01	2.42	0.32
Glycine	1.65	1.46	0.18
Histidine	0.61	0.45	0.07
Isoleucine	1.34	1.21	0.14
Leucine	2.54	2.38	0.28
Lysine	1.61	1.29	0.15
Methionine	0.06	0.07	0.01
Phenylalanine	1.49	1.35	0.16
Proline	1.72	1.56	0.19
Serine	1.70	1.42	0.19
Tryptophan	ND	ND	ND
Tyrosine	0.37	0.34	0.04
Threonine	1.63	1.45	0.19
Valine	2.03	1.82	0.21
Glutamine	ND	ND	ND
Asparagine	ND	ND	ND
Amino acid distribution (%)			
Nonpolar or hydrophobic	14.50	15.01	14.08
Uncharged polar	22.92	22.36	22.18
Negatively charged	32.93	32.06	34.51
Positively charged	23.08	24.27	22.54

ET1 (pH 10, 25 kHz, 400 W, 30 min, 50 °C, and *B. subtilis* proteases); ET2 (pH 10, 25 kHz, 400 W, 30 min, 50 °C, and *B. amyloliquefaciens* proteases); and ET3 (pH 10, 25 kHz, 400 W, 30 min, 50 °C, and *B. licheniformis* proteases).

**Table 4:** Effect of integrated extraction techniques on radical-scavenging activity, chelating capacity, and reducing power of *Brosimum alicastrum* Swartz nut proteins.

Extraction	In Vitro Antioxidant Properties (%)		
	Radical Scavenging	Chelating Capacity	Reducing Power
ET1	33.19 ± 2.5 ^a^	25.78 ± 2.8 ^b^	85.31 ± 4.1 ^c^
ET2	42.56 ± 1.7 ^b^	18.73 ± 1.1 ^a^	73.08 ± 2.6 ^b^
ET3	32.16 ± 1.8 ^a^	52.94 ± 5.7 ^c^	53.43 ± 3.1 ^a^

ET1 (pH 10, 25 kHz, 400 W, 30 min, 50 °C, and *B. subtilis* proteases); ET2 (pH 10, 25 kHz, 400 W, 30 min, 50 °C, and *B. amyloliquefaciens* proteases); and ET3 (pH 10, 25 kHz, 400 W, 30 min, 50 °C, and *B. licheniformis* proteases). Sample protein concentration = 100 mg/mL. Media ± standard deviation (n = 3). Different letters indicate significant differences (*p* < 0.05).

**Table 5:** Effect of integrated extraction techniques on inhibition of protein thermal denaturation and cell membrane stabilization of *Brosimum alicastrum* Swartz nut proteins.

Extraction	In Vitro Anti-Inflammatory Properties (%)	
	Inhibition of Protein Thermal Denaturation	Cell Membrane Stabilization
ET1	40.44 ± 3.5 ^a^	4.78 ± 0.8 ^a^
ET2	64.11 ± 4.7 ^c^	7.73 ± 1.1 ^a^
ET3	57.35 ± 2.8 ^b^	5.94 ± 0.6 ^b^

ET1 (pH 10, 25 kHz, 400 W, 30 min, 50 °C, and *B. subtilis* proteases); ET2 (pH 10, 25 kHz, 400 W, 30 min, 50 °C, and *B. amyloliquefaciens* proteases); and ET3 (pH 10, 25 kHz, 400 W, 30 min, 50 °C, and *B. licheniformis* proteases). Sample protein concentration = 100 mg/mL. Media ± standard deviation (n = 3). Different letters indicate significant differences (*p* < 0.05).

## Data Availability

The original contributions presented in the study are included in the article, further inquiries can be directed to the corresponding author.

## References

[1] Tay W., Quek R., Lim J., Bhupinder K., Shalini P., Christiani J.H. (2023). Plant-based alternative proteins—Are they nutritionally more advantageous?. Eur. J. Clin. Nutr..

[2] Santillán-Fernández A., González-Pérez C., Bautista-Ortega J., Huicab-Pech Z.G., Escobar-Castillo J., Larqué-Saavedra A. (2020). *Brosimum alicastrum* Swartz como alternativa para la reconversión productiva de áreas agrosilvopastoriles en Campeche. Rev. Mex. Cienc. For..

[3] Tang J., Yao D., Xia S., Cheong L.Z., Tu M. (2024). Recent progress in plant-based proteins: From extraction and modification methods to applications in the food industry. Food Chem. X.

[4] Chibuye B., Singh S.I., Chimuka L., Maseka K.K. (2023). A review of modern and conventional extraction techniques and their applications for extracting phytochemicals from plants. Sci. Afr..

[5] Muhammed N., Kappat V.S., Basheer A., Cherakkathodi S., Plachikkattu P.A., Shabir A.M., Nemtanu M.R., George J., Lackner M., Mousavi K.A. (2023). Contemporary insights into the extraction, functional properties, and therapeutic applications of plant proteins. J. Agric. Food Res..

[6] Anuruddika H., Oladipupo O.O., Chamila N., Maneka M., Rotimi E.A., Nandika B. (2022). Novel extraction technologies for developing plant protein ingredients with improved functionality. Trends Food Sci. Technol..

[7] Olatunde O.O., Owolabi I.O., Fadairo O.S., Ghosal A., Coker O.J., Soladoye O.P., Aluko R.E., Bandara N. (2023). Enzymatic modification of plant proteins for improved functional and bioactive properties. Food Bioprocess Technol..

[8] Rodríguez-Ambriz S.L., Martínez-Ayala A.L., Millán F., Dávila-Ortíz G. (2005). Composition and functional properties of Lupinus campestris protein isolates. Plant Foods Hum. Nutr..

[9] Bradford M.M. (1976). A rapid and sensitive method for the quantitation of microgram quantities of protein utilizing the principle of protein-dye binding. Anal. Biochem..

[10] Pedroche J., Yust M., Lqari H., Giron-Calle J., Alaiz M., Vioque J., Millan F. (2004). *Brassica carinata* protein isolates: Chemical composition, protein characterization and improvement of functional properties by protein hydrolysis. Food Chem..

[11] Sze-Tao K., Sathe S. (2000). Functional properties and in vitro digestibility of almond (*Prunus dulcis* L.) protein isolate. Food Chem..

[12] Kaur M., Singh N. (2005). Studies on functional, thermal, and pasting properties of flours from different chickpea (*Cicer arietinum* L.) cultivars. Food Chem..

[13] Diniyah N., Alam B.M., Lee S.-H. (2020). Antioxidant potential of non-oil seed legumes of Indonesian’s ethnobotanical extracts. Arab. J. Chem..

[14] Niveditha V.R., Sridhar K.R. (2014). Antioxidant activity of raw, cooked and Rhizopus oligosporus fermented beans of Canavalia of coastal sand dunes of Southwest India. J. Food Sci. Technol..

[15] Khan A., Khan H., Rauf A., Ben Hadda T. (2015). Inhibition of thermal induced protein denaturation of extract/fractions of Withania somnifera and isolated withanolides. Nat. Prod. Res..

[16] Yesmin S., Paul A., Naz T., Atiqur Rahman A.B.M., Akhter S.F., Ibne Wahed M.I., Emran T.B., Siddiqu S.A. (2020). Membrane stabilization as a mechanism of the anti-inflammatory activity of ethanolic root extract of Choi (*Piper chaba*). Clin. Phytosci..

[17] Grossmann L., David Julian McClements D.J. (2023). Current insights into protein solubility: A review of its importance for alternative proteins. Food Hydrocoll..

[18] Zhang X., Wang Q., Liu Z., Zhi L., Jiao B., Hu H., Ma X., Agyei D., Shi A. (2023). Plant protein-based emulsifiers: Mechanisms, techniques for emulsification enhancement and applications. Food Hydrocoll..

[19] Kyriakopoulou K., Keppler J.K., van der Goot A.J. (2021). Functionality of ingredients and additives in plant-based meat analogues. Foods.

[20] Del Mar Contreras M., Lama-Muñoz A., Gutiérrez-Pérez J.M., Espínola F., Moya M., Castro E. (2019). Protein extraction from agri-food residues for integration in biorefinery: Potential techniques and current status. Bioresour. Technol..

[21] Kumar M., Tomar M., Potkule J., Verma R., Punia S., Mahapatra A., Belwal T., Dahuja A., Joshi S., Berwal M.K. (2021). Advances in the plant protein extraction: Mechanism and recommendations. Food Hydrocoll..

[22] Görgüç A., Özer P., Yılmaz F.M. (2020). Simultaneous effect of vacuum and ultrasound assisted enzymatic extraction on the recovery of plant protein and bioactive compounds from sesame bran. J. Food Compos. Anal..

[23] Di Filippo G., Melchior S., Plazzotta S., Calligaris S., Innocente N. (2024). Effect of enzymatic hydrolysis with Alcalase or Protamex on technological and antioxidant properties of whey protein hydrolysates. Food Res. Int..

[24] Ahmadinejad F., Geir-Møller S., Hashemzadeh-Chaleshtori M., Bidkhori G., Jami M.S. (2017). Molecular mechanisms behind free radical scavengers function against oxidative stress. Antioxidants.

[25] Kumar M., Tomar M., Punia S., Dhakane-Lad J., Dhumal S., Changan S., Senapathy M., Berwal M.K., Sampathrajan V., Sayed A.S. (2022). Plant-based proteins and their multifaceted industrial applications. LWT.

[26] Langyan S., Yadava P., Khan F.N., Dar Z.A., Singh R., Kumar A. (2022). Sustaining protein nutrition through plant-based foods. Front. Nutr..

[27] Seelig J., Seelig A. (2023). Protein Stability-Analysis of heat and cold denaturation without and with unfolding models. J. Phys. Chem. B.

